# Photoreceptor physiology of two species of crab spiders (Araneae: Thomisidae)

**DOI:** 10.1007/s00359-026-01802-8

**Published:** 2026-03-24

**Authors:** Frederik Leck Fischer, Nikolaj Scharff, Anders Garm

**Affiliations:** 1 Marine Biological Section, University of Copenhagen, Universitetsparken 4, Copenhagen Ø, 2100 Copenhagen, Denmark; 2 Natural History Museum Denmark, University of Copenhagen, Universitetsparken 15, 2100 Copenhagen, Denmark

**Keywords:** Eye physiology, Vision, Sensory ecology, Spiders, Thomisidae, *Xysticuscristatus*, *Ozyptilapraticola*, Araneae

## Abstract

Spiders are a diverse order of predatory arachnids with more than 53.000 described species, most of which have eight eyes. Many webless hunting spiders, most noticeably the jumping spiders (Salticidae) have been shown to have excellent eyes with high spatial resolution and colour vision. The family of crab spiders (Thomisidae) is also hypothesized to be visual hunters, employing a “sit and wait” or ambush hunting technique; however, little is currently known about their visual capacity. Here we use extracellular electrophysiology to examine the photoreceptor physiology of two crab spiders living in two different ecological niches. *Ozyptila praticola* (C.L. Koch, 1837) hunts on the ground in dim habitats whereas *Xysticus cristatus* (Clerck, 1757) hunts in the typical bright open grasslands. We test the hypotheses that (1) each species has special-purpose eyes, (2) that males and females have different photoreceptor physiology based on observed differences in behavior, and (3) the niche difference of the two species is reflected in different visual properties. We found support for the first hypothesis and also some support for differences between the two species, mostly in the temporal resolution of the eyes. We found no evidence of differences between males and females, indicating that the two sexes exhibit more shared visually guided behaviors than previously thought.

**Supplementary Information:**

The online version contains supplementary material available at 10.1007/s00359-026-01802-8.

## Introduction

Spiders are known for their visual system, with the majority containing 4 pairs of camera-type eyes. Vision has been studied in depth in relatively few species of spiders, but the results show great variation across taxa. In spiders, vision is used in important behavioral tasks such as mate recognition, navigation, and predator and prey detection (Barth 2002; Foelix 2011; Norgaard et al. 2008; Wilson et al. 2024).

Vision is especially important for actively hunting spiders. Of these, jumping spiders (Salticidae) have been the subject of most studies and they have superb vision for animals of their size in terms of spatial resolution, color vision (with a tiered retina and spectral filters) and depth perception (Land 1969a, b; Eakin and Brandenburger 1971; Blest et al. 1981). Spiders of the families Gnaphosidae and Lycosidae are also actively hunting spiders that have evolved the ability to detect polarized light, which they use during homing and navigation (Dacke et al. 1999; Ortega-Escobar and Munoz-Cuevas 1999). Representatives of the spider families Trechaleidae, Deinopidae and Sparassidae are nocturnal but still rely on vision when hunting and navigating at night. The high sensitivity of these nocturnal spiders’ eyes is due to adaptations like large lenses, long integration times, enlarged rhabdoms, and the presence of a tapetum lucidum at the back of the secondary eyes (Blest 1977; Blest et al. 1978; Barth et al. 1993; Pirhofer-Walzl et al. 2007; Norgaard et al. 2008; Benson and Suter 2013). Like in other visually guided animals, the adaptations of the visual system of spiders are intricately linked to their specific niche and life strategy, which has enabled researchers to directly use examination of their visual system to explain observed behavior (Norgaard et al. 2008; Ortega-Escobar 2011; Insausti et al. 2012; Ortega-Escobar and Ruiz 2017). There has, however, been a strong bias in the choice of study animals, most noticeably towards the jumping spiders and the wandering spider *Cupiennius salei* (Keyserling, 1877), and the eyes of many other spider families remain understudied.

Crab spiders of the family Thomisidae are also actively hunting spiders (Foelix 2011), and most are ambush/sit-and-wait predators in the vegetation or on the ground (Bristowe 1958; Morse 1984). Many have large raptorial forelegs and hunt by slowly sneaking up on unsuspecting prey while orienting their body so their widely spaced anterior eyes are directed at the prey (pers. observations). The only crab spider where the visual capacity has been examined to some depth is *Misumena vatia* (Clerck, 1757). The retinas of the anterior median eyes of *M. vatia* are tiered into three layers of different rhabdoms not unlike those used in color vision in jumping spiders (Land 1969b; Insausti et al. 2012) and in line with this, a study found evidence of at least two opsins being expressed in all eyes of *M. vatia* (Defrize et al. 2011). One with a sensitivity peak in the UV region (around 340 nm) and one most sensitive to green light (around 520 nm). It also has an overlapping field of view across multiple eyes, allowing for the combination of information from multiple visual systems (Insausti et al. 2012).

To deepen our understanding of the visual capacity of crab spiders, we have examined the visual physiology of *Xysticus cristatus* (Clerck, 1757) and *Ozyptila praticola* (C. L. Koch, 1837), two of the most common and readily available species of crab spiders native to temperate Europe. They are both sit-and-wait predators but hunt in different niches. The one species, *X. cristatus*, is active in daytime, and is often found in the vegetation in grassland and similar open, sunny, and at times windy habitats (Nielsen 1932). It primarily preys on large day-active prey such as hoverflies, bees, beetles, crane flies, and occasionally also ants (Nielsen 1932; Bristowe 1958; Foelix 2011; Own observations) (Fig. S1). The other species, *O. praticola*, is a slightly smaller species, well-camouflaged and cryptic. It is usually found on the ground in dark habitats, hidden under leaves, stones, bark of trees, or under similar organic debris (Nielsen 1932; Own observations). It has been observed feeding on a range of small, slow-moving animals like springtails (Collembola), pseudoscorpions, and small beetles abundant in the same habitat (Fig. S2). Both species also display sexual dimorphism, especially seen as differences in size and behavior with the male being smaller and more actively moving around searching for females. Supplementing our central electrophysiological experiments examining different visual parameters (absolute sensitivity, temporal resolution, and spectral sensitivity) from all four sets of eyes with field observations of behavior and ecology, we aim to test the following three hypotheses:

1. The four eye types of each species will have different purposes/capabilities, as seen in other spiders, and it will be reflected in differences in the eyes’ physiology.
2. The behavioral differences observed between males and females are reflected in the visual system.
3. The photoreceptor physiology of the two species will align with their respective niches.

## Materials and methods

### Animals

Live specimens were collected on several occasions from different locations during the spring/summer of 2022 and 2023. Males were primarily collected at the end of May and the start of June when mature, and females were collected throughout the summer.

Grassland habitats in Northern Zealand ([56.0575ºN, 12.5451ºE], [55.8212ºN, 12.4435ºE], [55.8234ºN, 12.5126ºE], [55.9706ºN, 11.8790ºE]) supplied the majority of *Xysticus cristatus* sampled with a sweep-net in the lower vegetation.

Females of *O. praticola* were mostly found in an urban green area in Copenhagen ([55.6350ºN, 12.5357ºE]. They were found under rocks and pieces of wood, and by sifting leaf litter. Males of *O. praticola* were collected in The Botanical Garden in the center of Copenhagen ([55.6861ºN, 12.5726ºE]), the coast of Northern Zealand ([55.9829ºN, 11.8752ºE]) and at Vestamager ([55.6182ºN, 12.5754ºE]).

**Fig. 1:**
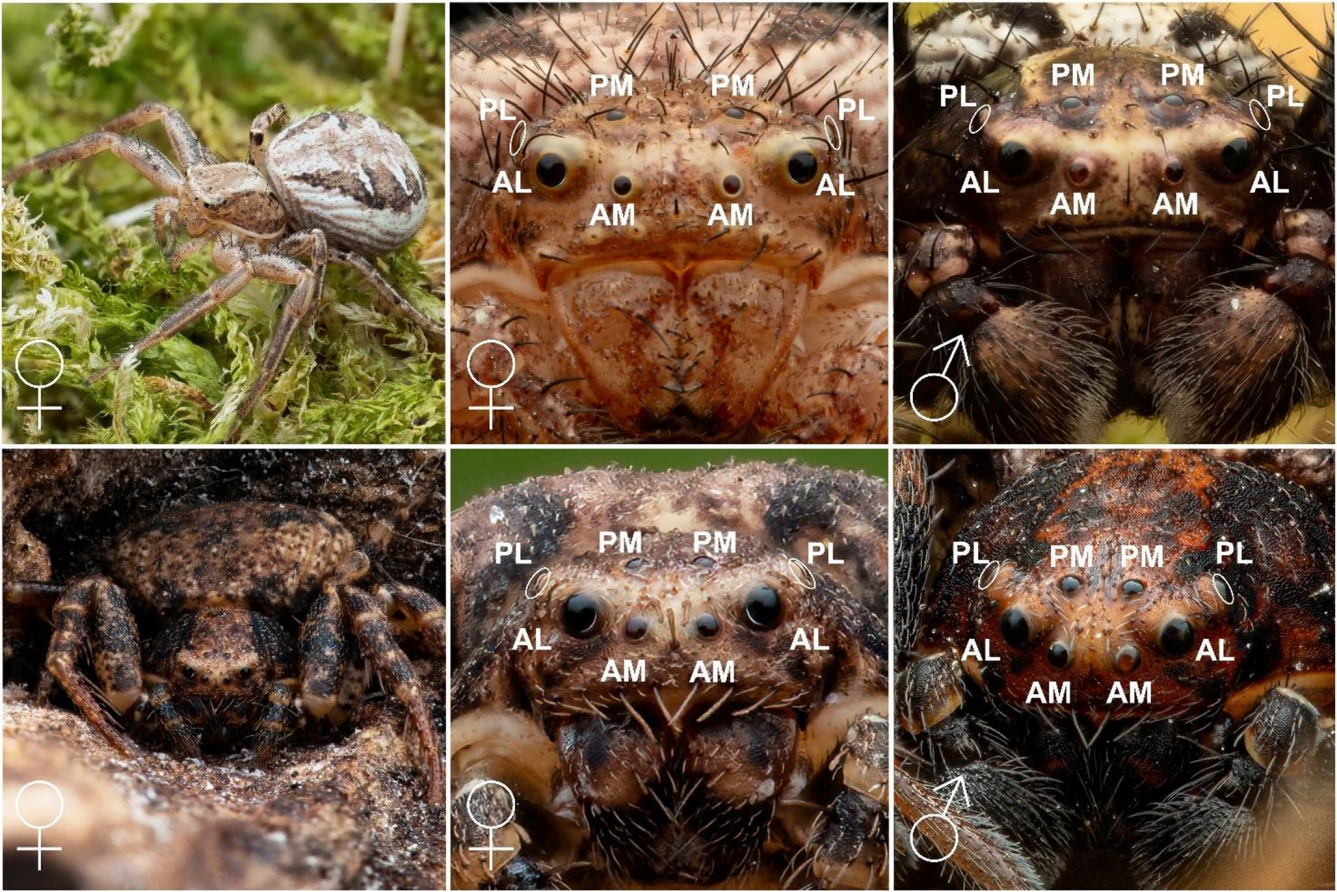
The experimental animals. Top row: *Xysticus cristatus*, a common day-active crab spider in Danish grasslands. Bottom row: *Ozyptila praticola*, a common crab spider in leaf litter under stones and in similar dim environments. *AM* anterior median eye, *AL* anterior lateral eye, *PM* posterior median eye, *PL* posterior lateral eye

Collected specimens of both species were kept in small plastic vials and supplied with humidity when deemed necessary (*Xysticus cristatus* can die quickly if kept in a vial with too high humidity). Live specimens of both species were photographed before the experiments (Fig. 1). After size measurements, species determination was checked by genital examination of the pedipalps and epigyne under a stereo microscope.

### Extracellular electrophysiology: electroretinograms

**Fig. 2:**
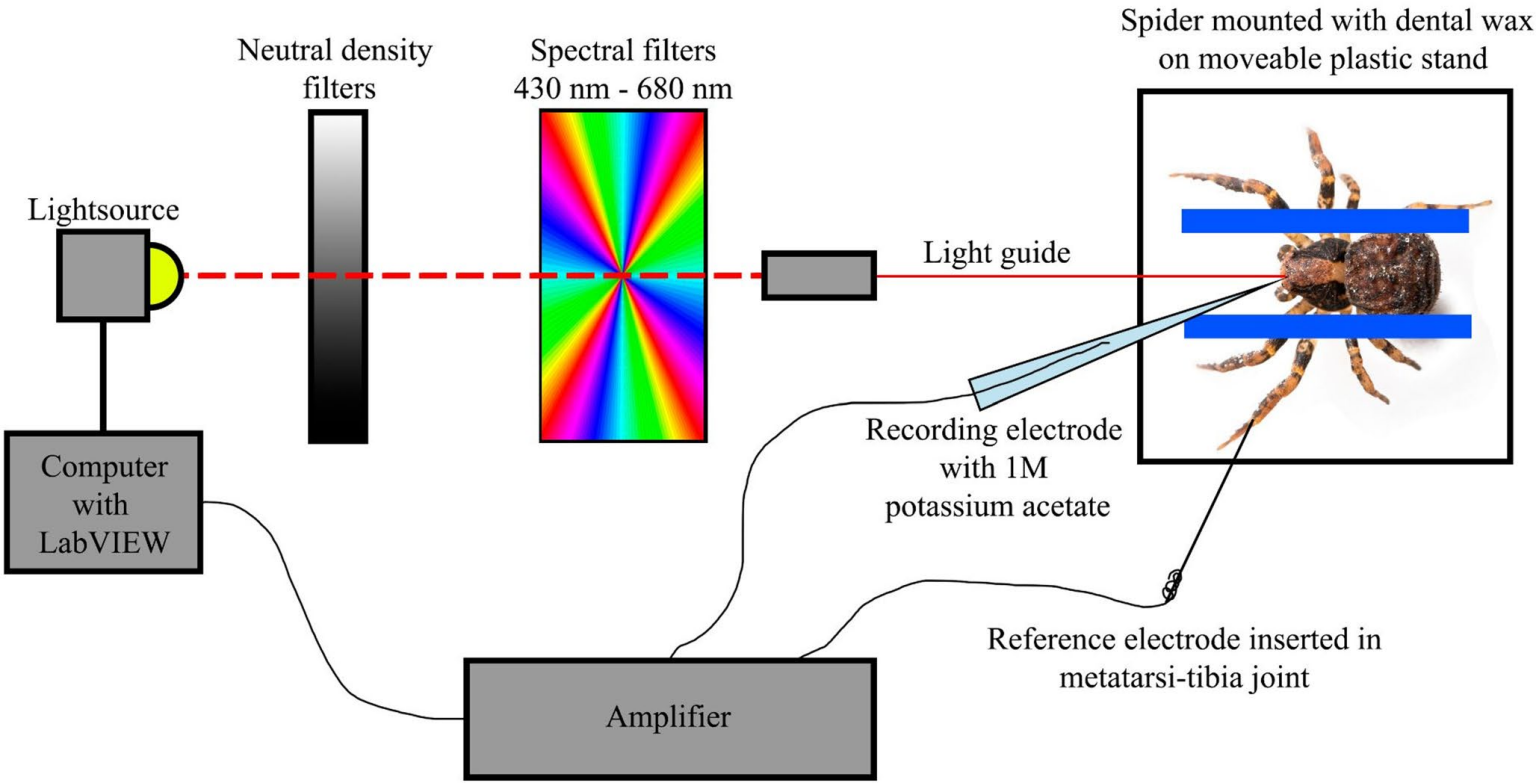
The experimental setup. A sedated crab spider is mounted on a plastic stand with dental wax. Visual stimuli is delivered to one eye type at a time with a light-guide. The light source is controlled by a computer and the type of visual stimuli is modified further by filters (depending on the experiment). Two electrodes (a recording electrode and reference electrode) records activity in the spider. The recordings are amplified and saved on a computer for further analysis

Live specimens were sedated with CO_2_ in plastic bags for 2–7 min, depending on the specimen. Most, however, were sufficiently sedated after 2 min. The sedated spider was quickly transferred to a small plastic stand where its legs were extended and fastened with small pieces of dental wax to restrict movements (Fig. 2). Usually, the abdomen was also held in place by dental wax, and a small piece of wax was placed in front of the pedipalps to restrict their movement. The plastic stand was then mounted in the electrophysiological setup. A custom-made recording glass electrode was placed in direct contact with the eye of interest and filled with 1 M potassium acetate as the electrolyte. Electrodes with different tips and pore sizes were used during the experiments, depending on eye size. The reference electrode (a thin wolfram needle soldered to a silver wire) was inserted in one of the legs, usually in the metatarsal-tibial joint of the second pair of legs (Fig. 2).

An ultra-bright LED (Luminus CBT-90 LED, Sunnyvale, CA, USA) was used as a source of white light stimuli, and the light was administered to the eyes using a small quartz light guide, with a diameter of 50 μm, placed directly in front of the eye. The exact angle of the light guide was decided based on the approximate center of the eye’s cuticular lens. Maximum intensity was 9 × 10^3^ W/(sr·m^2^) measured at the tip of the light guide (spectrum = 400–800 nm, X1.5 optometer, Gigahertz-Optik, Germany).

Initially, the average time for full dark adaptation of each eye was assessed. The specimens were left in darkness, and recordings were made approximately every 5 min using a standard stimulus (25 ms of maximum intensity). All eyes, in both species and both sexes, showed no or little changes in response after 40 min, which was then chosen as the duration of the dark adaptation for all experiments. Experiments were conducted between 9 am and 11 pm. Recordings done at noon showed similar responses as those done in the evening or in the morning, indicating that there is no circadian rhythm potentially affecting the results.

All recordings were amplified 1000 times using a DC1700 differential amplifier (A-M Systems, Sequim, WA, USA). Background noise was reduced using an internal filter in the amplifier (50 Hz notch filter, a 0.1 Hz high-pass filter, and a 1 kHz low-pass filter). The recordings were digitized at 1 kHz using an NI USB-6229 DAQ card (National Instruments, Austin, TX, USA). A custom LabVIEW program (National Instruments) controlled all the experiments.

### Absolute sensitivity, *V*-Log *I* curves

The eye’s absolute sensitivity was tested using pulse stimulations of white light at varying intensities. Stimulations started at the lowest light intensity with no response, and the intensity was then increased in steps of 0.3 or 0.7 log units using neutral density filters (Linos, Goettingen, Germany) with 1 min between stimulations (Fig. 2). The duration of the stimulus was 15 ms for the anterior lateral eyes, as these were found to be generally faster in both species in the test runs done before experiments, and 25 ms for all other eyes. The absolute intensity span covered six log units from 0.09 W/(sr^−^·m^2^) to 9000 W/(sr·m^2^). (See fig. S3 for examples). All recordings were manually analyzed using the software Igor Pro (Version 9.0.1.2. WaveMetrics, Inc., Oregon, USA). The amplitude was read for each stimulation and normalized setting the maximum amplitude in each recording series to 1. The normalized data were used to make *V*-log *I* curves, and 95% confidence intervals were calculated using the “CONFIDENCE”-feature in Microsoft Excel. Since males and females showed no significant difference in either species (Fig. S5), the data from the two sexes were pooled to increase the n-value for each eye type of each species.

### Spectral sensitivity

The eye’s spectral sensitivity was tested using light flashes of the same duration as for the absolute intensity, with 1 min between stimulations. The light stimuli were filtered by interference colour filters (half width = 12 nm, CVI Laser, Bensheim, Germany) covering 430 nm to 680 nm in steps of 10 or 20 nm (Fig. 2). The intensity of the stimuli was adjusted to achieve an equal quanta stimulation of 1.7 × 10^19^ photons s^− 1^· sr^− 1^· m^− 2^ at each wavelength. Another 2nd *V*-log *I* curve was done after the spectral series to make sure that the eye had not changed its sensitivity.

All recordings were manually analyzed using the software Igor Pro (Version 9.0.1.2, WaveMetrics, Inc. Oregon, USA). To make a spectral sensitivity curve (showing the probability of photon absorption at different wavelengths), the amplitude of all the recordings was transformed using the same eye’s *V*-log *I* curve (Coates et al. 2006). If multiple values of the second test *V*-log *I* curve were more than 10% different from the first *V*-log *I* curve, all response amplitudes to the colour stimuli were corrected assuming a time-wise linear change in sensitivity. Of 31 eyes tested, 5 eyes were subjected to this procedure.

The transformed spectral recordings were normalized, setting the maximum response in each series to 1, and 95% confidence intervals were calculated using the “CONFIDENCE”-feature in Excel. The response peak in all analyzed eyes of both males and females fell within 480–530 nm, a range considered possible evidence of a shared opsin across all analyzed eye types. This was the basis for pooling them and increasing the n value for each species. The obtained spectral curves were compared to theoretical opsin curves (Govardovskii et al. 2000) using the least square of the mean method, to find the best possible fit to a single opsin.

### Temporal resolution, flicker fusion frequency (FFF)

Each eye’s flicker fusion frequency (FFF) was tested after the absolute and spectral sensitivity had been tested. Initially, the eye was light-adapted at mid intensity (4.5 × 10^3^ W/(sr^·^ m^2^) for 5 min. The eye was then subjected to a series of 10 s sinusoidal flickering stimuli with 20 s constant light at mid intensity between them (See fig. S4 for examples of recordings). The starting frequency, the number of frequencies tested, and the frequency interval varied depending on eye type and species. These settings were determined during a “test run” for each eye/species. If the eye still had clear responses at 20 Hz, a higher starting frequency and increment steps of 5 Hz were chosen. Similarly, if the eye’s maximum response was less than 20 Hz, a starting frequency of 2 or 2,5, and increments of 2 or 2,5 Hz were chosen.

All recordings were manually analyzed using the software Igor Pro (Version 9.0.1.2, WaveMetrics, Inc., Oregon, USA). Each recording went through a Fourier transformation of a time span matching 10 cycles of stimulations (10 s at 1 Hz, 1 s at 10 Hz, etc.). The power of the principal frequency (the frequency of the given stimulation) would be used for the flicker fusion frequency curve. These values were normalized, setting the maximum response in each series to 1, and the 95% confidence intervals were calculated using the “CONFIDENCE”-feature in Excel. Since it is based on ERG recordings, the FFF cannot be determined by the internal noise of the photoreceptors, and instead 5% of the maximum response was chosen as a conservative estimate of the flicker fusion frequency. Males and females showed no obvious differences in their FFF-curves in any of the eye types (Fig. S6), and data from the two sexes were pooled to increase the n values.

## Results

### Eye placement and size

The anterior lateral (AL) eyes are the largest in both species (See Table 1). They have a wide spacing between them and are directed forward. The anterior median (AM) eyes are small in both species. They are located between the AL eyes. The retina of the AM eyes is moveable, and was seen moving in both species while mounting the lightguide for the experiments. The posterior median (PM) eyes are directed upwards and are the smallest eyes, together with the anterior median eyes. The posterior lateral (PL) eyes are directed diagonally backwards, with a wide spacing.

**Table 1:** Measurements of eye sizes of 3 males and 3 females of *Xysticus cristatus* and *Ozyptila praticola *in mm

	AL	AM	PL	PM
*Xysticus cristatus*				
Female 1	0.132	0.075	0.106	0.081
Female 2	0.13	0.082	0.102	0.073
Female 3	0.148	0.085	0.118	0.093
Male 1	0.133	0.072	0.094	0.085
Male 2	0.128	0.086	0.108	0.085
Male 3	0.134	0.08	0.1	0.083
*Ozyptila praticola*				
Female 1	0.117	0.071	0.085	0.073
Female 2	0.115	0.068	0.091	0.068
Female 3	0.12	0.072	0.085	0.061
Male 1	0.122	0.074	0.092	0.065
Male 2	0.122	0.085	0.097	0.076
Male 3	0.136	0.085	0.099	0.071

*AM* anterior median eye, *AL* anterior lateral eye, *PM* posterior median eye, *PL* posterior lateral eye

Eye sizes were not found to be significantly different between sexes in *Xysticus cristatus* (unpaired 2-tailed t-test with equal variance, 0.27 < *p* < 0.80). Both AM and PL eyes were significantly larger in *O. praticola* males than in females, despite a smaller body size, although in AM the value is borderline significant and should be interpreted with caution (See Table 2).

**Table 2:** Results from two-tailed t-tests of the difference in eye sizes between sexes of *Ozyptila praticola*, and *Xysticus cristatus*

*Xysticus*	AL	AM	PL	PM
P-value	0.450877287	0.803753802	0.272294037	0.76428082

*Ozyptila*	AL	AM	PL	PM
P-value	0.128807629	**0.046359897**	**0.035605984**	0.518518519

*AM* anterior median eye, *AL* anterior lateral eye, *PM* posterior median eye, *PL* posterior lateral eye, **bold = P<0.05**

### Absolute sensitivity and dynamic range, *V*-Log *I*

There was no evidence of differences between male and female *V*-Log *I* curves of either species with all examined eyes (Fig. S5). The sexes were therefore pooled in the subsequent analyses.

**Fig. 3:**
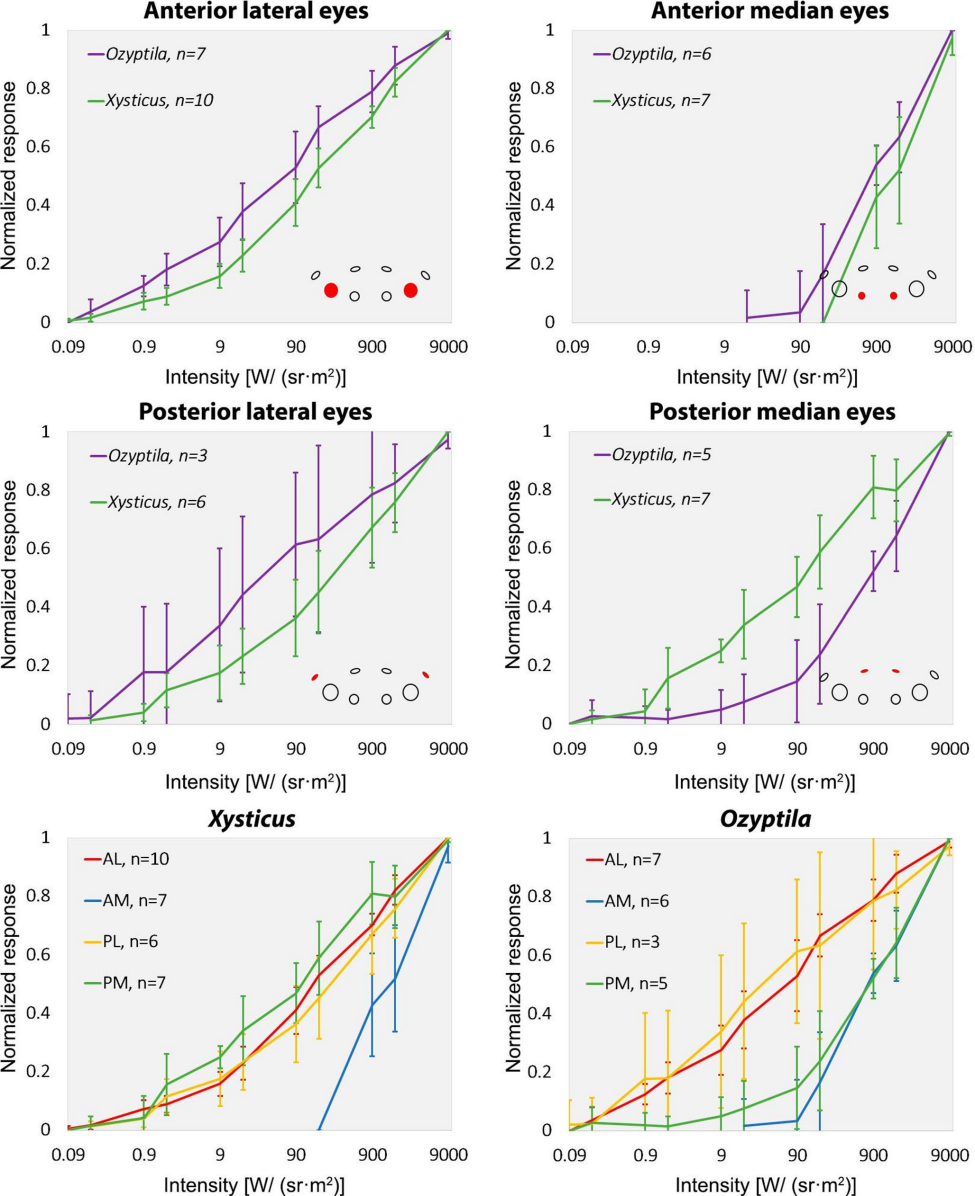
Absolute sensitivity - *V*-log curves. Top and center row: Comparison of normalized *V*-log *I* curves of eyes in *Xysticus cristatus* and *Ozyptila praticola*. Except for the PM eye, there is a tendency for *O. praticola* being more sensitive, with curves being skewed more to the left. Bottom row: Comparison of all four eyes’ *V-*log *I* curves within each species. Both species have two categories of eyes in regard to sensitivity. X-axis is log-transformed. Error bars indicate 95% confidence intervals. *AM* anterior median eye, *AL* anterior lateral eye, *PM* posterior median eye, *PL* posterior lateral eye

The *V*-Log *I* curves of the AL eyes were based on ten *X. cristatus*, (5 males and 5 females) and seven *O. praticola* (3 males, 4 females). The AL eye shows a broad dynamic range of at least 3 log units in *X. cristatus* and a sensitivity threshold around 9 W/(sr ·m^2^)^,^ and a dynamic range of at least 4 log units in *O. praticola* and a sensitivity threshold around 0.9 W/(sr·m^2^) (Fig. 3). The dynamic range is defined as the approximately linear part of the *V*-log *I* curve, and the sensitivity threshold is defined as the last point of the linear part of the *V*-log *I* curve. There is a slight overlap of the 95% confidence intervals at most points. Neither curve saturates at maximum intensity, indicating a broader dynamic range than recorded for both species.

The *V*-Log *I* curves of the AM eyes are based on seven *X. cristatus* (3 males and 4 females), and six *O. praticola* (3 males, 3 females). The AM eyes’ dynamic range is at least 2 log units for *O. praticola*, and the sensitivity threshold is approximately 90 W/(sr·m^2^) and at least 1.7 log units for *X. cristatus* with a sensitivity threshold between 180 W/(sr·m^2^) and 900 W/(sr·m^2^). Neither curve saturates at maximum intensity, indicating a broader dynamic range than recorded for both species (Fig. 3). The 95% confidence intervals of the two curves overlap at higher intensities, but not at intensities lower than 900 W/(sr·m^2^), indicating that the AM eyes of *O. praticola* seems to be a bit more sensitive than in *X. cristatus.*

The *V*-Log *I* curves from PL eyes are based on six *X. cristatus* (3 males and 3 females) and three *O. praticola* (3 males). Three *O. praticola* females were also tested; however, the results were of too low quality and were excluded from the analysis. The recordings from *O. praticola* males were noisy (large 95% confidence intervals). The dynamic range is at least 3 + log units and the sensitivity threshold is around 9 W/(sr·m^2^) in *X. cristatus* and at least 4,7 + log units with a threshold around 0,0.18 W/(sr·m^2^) in *O. praticola* (Fig. 3). For both species, there is little evidence of saturation at maximum intensity, indicating a broader dynamic range than recorded for both species. There is a tendency for the PL eyes of *O. praticola* males being more sensitive with the *V*-log *I* curve shifted to the left.

The *V*-Log *I* curves of the PM eyes are based on eight *X. cristatus* (4 males and 4 females), and five *O. praticola* (2 males, 3 females). The dynamic range spans at least 4 + log units with the sensitivity threshold around 0.9 W/(sr·m^2^) in *X. cristatus* and at least 2 + log units and a threshold of 90 W/(sr·m^2^) in *O. praticola* (Fig. 3). There is no overlap of the 95% confidence intervals of the *V*-Log *I* curve at lower intensities, meaning the PM eye of *X. cristatus* is significantly more sensitive. For both species, there is no saturation at maximum intensity, indicating a broader dynamic range than measured.

The ALs, PLs, and PMs eyes of *Xysticus cristatus* all show very similar absolute sensitivities with *V*-log *I* curves overlapping in the 95% confidence intervals (Fig. 3). The AM eyes, however, are less sensitive than the other eyes and stop responding approximately at 2.3 log units higher intensity. In *Ozyptila praticola*, the PMs and AMs eyes are similar with absolute sensitivities around 90 W/(sr·m^2^). They differ from the ALs and PLs eyes which are both more sensitive, and there is only a little overlap of confidence intervals with the PMs and AMs eyes. Note that, for *O. praticola* PL eyes, data were only obtained from males.

### Temporal resolution, flicker fusion frequency (FFF)

There were no obvious differences between males and females of either species (Fig. S6), which again led us to pool the data from the two sexes together.

**Fig. 4:**
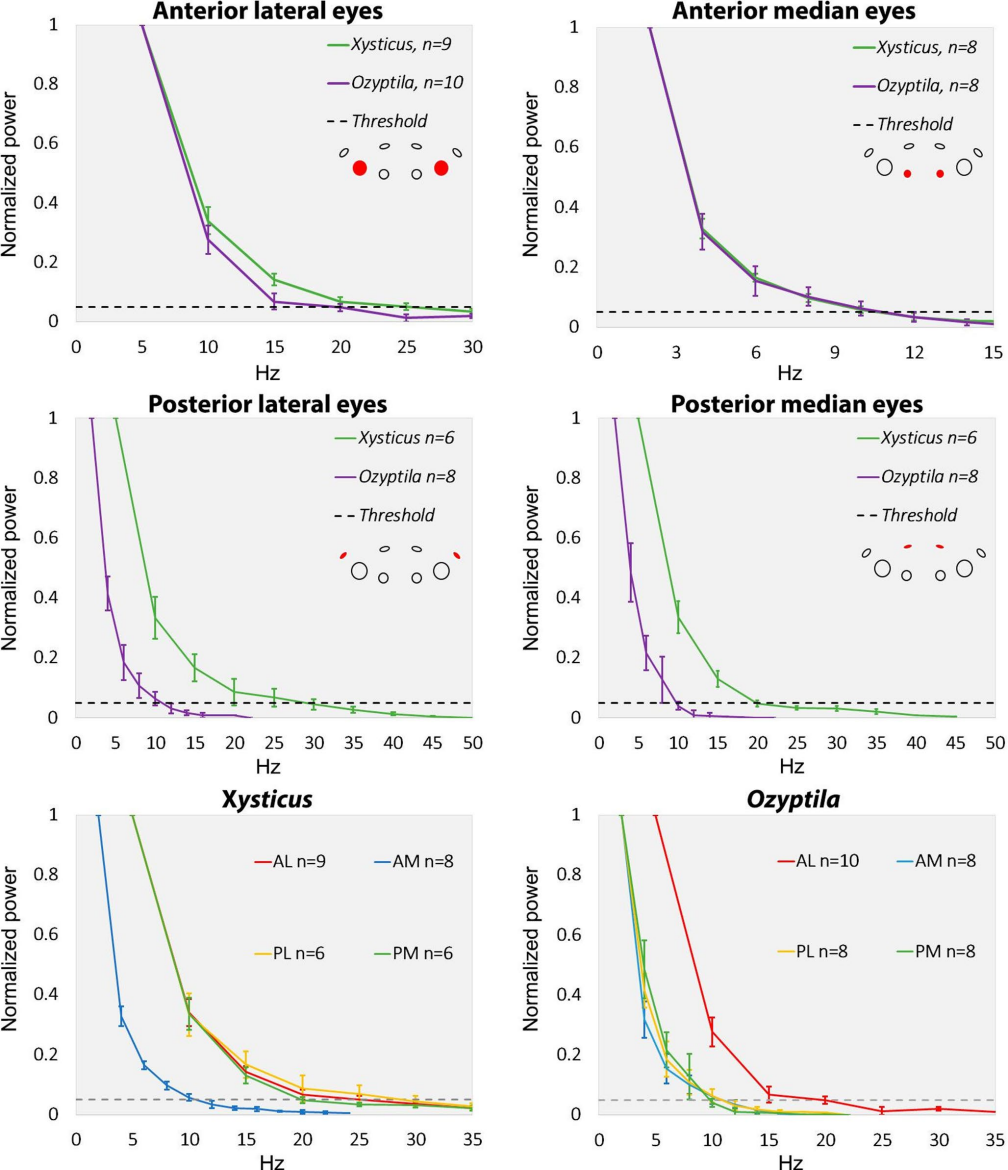
Temporal resolution. Top and center row: Temporal resolution measured as the flicker fusion frequency (FFF) in the eyes of *Xysticus cristatus* and *Ozyptila praticola* eyes. FFF is defined as 5% of the maximum power (dotted line). PL and PM showed large differences in their response between species, and as a result were recorded at different frequency increments (2 Hz in *O. praticola* & 5 Hz in *X. cristatus*). This allows for reading of the FFF but not a direct comparison. Bottom row: Comparison of eye types within a species, showing two clear categories of eyes in each species regarding the FFF. Error bars indicate 95% confidence intervals. *AM*: anterior median eye, *AL* anterior lateral eye, *PM* posterior median eye, *PL* posterior lateral eye

The FFF-curves of the AL eyes are based on nine *X. cristatus* (4 males and 5 females), and ten *O. praticola* (6 males, 4 females). The FFF (the frequency where the relative normalized response drops to 0.05) of the AL eye in *O. praticola* is around 19–20 Hz, while in *X. cristatus* it is around 25 Hz (Fig. 4). The AL eyes of the two species were recorded in similar frequency intervals, allowing for direct comparison. There are clear differences, with no overlap of the 95% confidence at 25 Hz (Fig. 4). Eight AM eyes of *X. cristatus* (4 males and 4 females), and eight *O. praticola* (4 males and 4 females) were used to test their temporal resolution. The AM eyes of the two species were recorded in similar frequency intervals, allowing for direct comparison, and in both cases, the FFF was just above 10 Hz, and their similar response is also seen by an overlap of the 95% confidence intervals for all data points. The FFF-curves of the PL eyes are based on six eyes for *X. cristatus* (3 males and 3 females), and eight eyes for *O. praticola* (6 males and 2 females). As the two species were recorded at different frequency intervals, a direct comparison of the curves is not possible; however the FFF has the largest difference of any eyes (Fig. 4). The FFF of the PL eye, in *O. praticola*, is again just above 11 Hz, and around 30 Hz in *X. cristatus*. Six PM eyes were used for the FFF-curves of *X. cristatus* (3 males and 3 females), and 8 *O. praticola* (5 males and 3 females). The difference in tested frequencies obscured a direct comparison; however, the FFF of the two species suggests a large difference (Fig. 4). The FFF of *O. praticola* PM eyes is around 10 Hz, while that of *X. cristatus* is around 20 Hz.

The AL, PL, and PM eyes of *X. cristatus* were recorded at similar Hz intervals allowing for direct comparison of graphs. These three pairs of eyes have very similar temporal resolution with largely overlapping 95% confidence intervals in the FFF-curves and similar high FFF between 20 and 30 Hz (Fig. 4). The AM differs from the other eyes, having a FFF of only about 10 Hz. In *O. praticola* AM, PM, and PL eyes had similar FFF curves with overlapping confidence intervals and similar FFF of approx. 10–12 Hz (Fig. 4). The AL are significantly faster eyes with a much higher FFF of 20 Hz.

### Spectral sensitivity

**Fig. 5:**
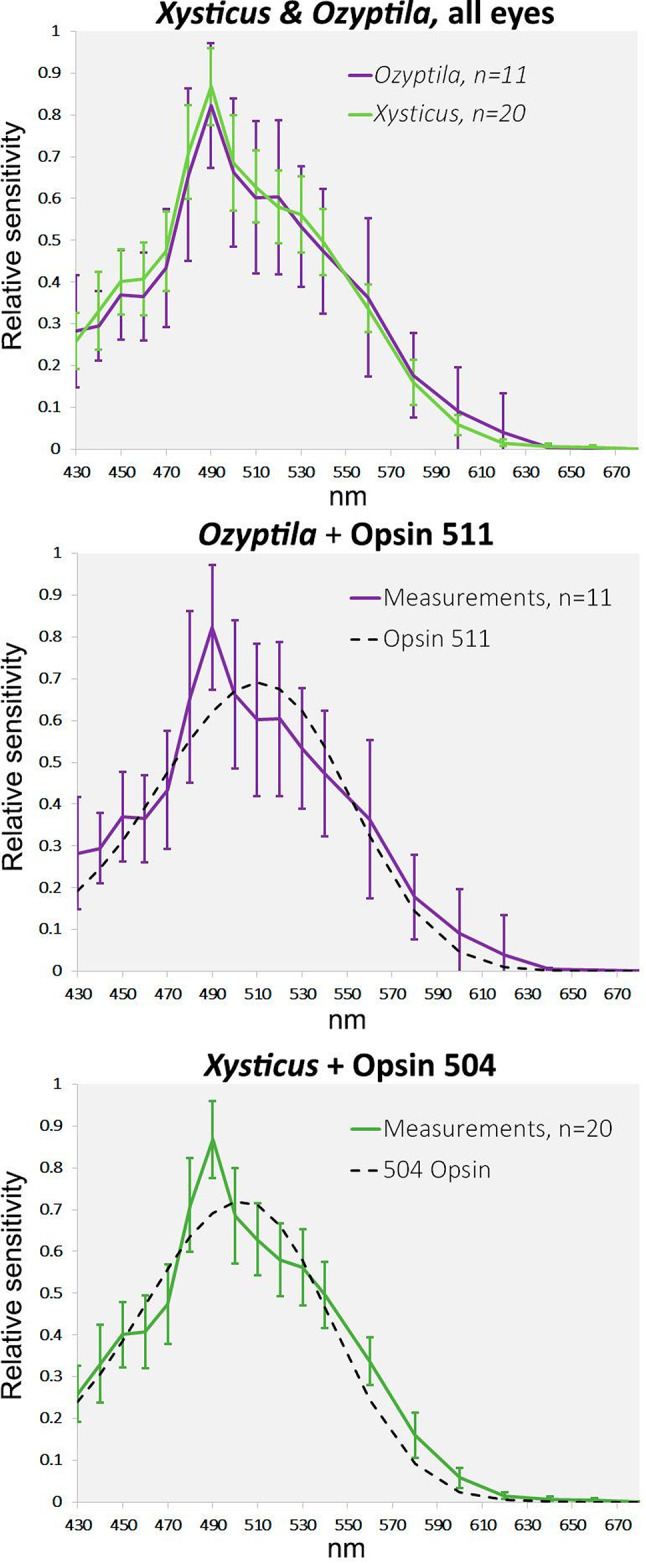
Spectral sensitivity. Top graph: Spectral sensitivity curve of *Xysticus cristatus* and *Ozyptila praticola* with data from all eyes types pooled together. Note the very similar curves with a single peak in the blue green part of the spectrum (490–500 nm). Center and bottom graphs: Solid lines indicate the spectral sensitivity of *O. praticola* and *X. cristatus*, respectively. Broken lines show the best fit (least squares method) to the theoretical absorption curve of a single opsin (Govardovskii et al. 2000). The best fit is to a 511 nm opsin in *O. praticola* and a 504 nm opsin in *X. cristatus*. Error bars indicate 95% confidence intervals

When analyzing each eye type separately, the spectral curves showed great variability (large 95% CI, see Fig. S7 for examples). However, they all had peaks in the blue-green part of the spectrum between 480 and 530 nm indicating that the eyes utilize the same or spectrally similar opsins. The presence of the same blue-green opsin across all eye types is also seen in another thomisid spider, *Misumena vatia* (Defrize et al. 2011), and in the wandering spider *Cupiennius salei* (Barth et al. 1993). Based on this and our results we assume that in both species the four eye types utilize the same blue-green opsin and we thus pooled all spectral data from each species, *n* = 20 for *X. cristatus* and *n* = 11 for *O. praticola*, to increase data quality (Fig. 5 top). The resulting curves from the two species were almost identical, with a single peak at 490 nm and relatively small 95% confidence intervals. Note that due to the relatively low intensity of the spectral stimuli, some of the eye types provided little data, and most data came from the large AL eyes (Table 3).

**Table 3:** Number of spectral sensitivity experiments done on each eye type of *Ozyptila praticola*, and *Xysticus cristatus*

Spectral sensitivity	AL	AM	PL	PM
*Ozyptila praticola *experiments	6	1	3	1
*Xysticus cristatus* experiments	9	1	5	5

*AM* anterior median eye, *AL* anterior lateral eye, *PM* posterior median eye, *PL* posterior lateral eye

The spectral curves for each species were compared to a theoretical opsin curve (Govardovskii et al. 2000) and the best fit was found using the least mean of the square method. The best fit for *O. praticola* is an opsin peaking at 511 nm, while the best fit of *X. cristatus* is a 504 nm opsin (Fig. 5 center & bottom).

## Discussion

We studied the photoreceptor physiology of two members of the spider family Thomisidae, *Ozyptila praticola* and *Xysticus cristatus*, to test the hypothesis that their different lifestyle is reflected in their visual ecology and thus in their photoreceptor properties. *O. praticola* is found in dim habitats at ground level, whereas *X. cristatus* is found in knee-high vegetation in open grassland. Our results supported the hypothesis, but interestingly, we found no differences between the sexes despite adult males and females have vastly different lifestyles in both species.

### Receptor physiology is similar in males and females

Our results showed that the eyes of males and females have similar receptor physiology in both examined species, despite their different behaviours. The females are more or less stationary and hidden, and the males are running around actively, in search of hidden females. This could indicate that they are both visually guided, like several other webless spiders (Land 1969a; Dacke et al. 1999; Norgaard et al. 2008), and/or visually detect the same type of predator, but more behavioral experiments are needed before any conclusion can be drawn. The similar physiology is also surprising since both the AM and PL eyes of the male *O. praticola* are larger than in females, which should lead to a higher photon capture and potentially higher sensitivity and/or higher temporal resolution (Lazareva et al. 2012). It could be explained by the males having higher spatial resolution, which typically also requires more photons (Land and Nilsson 2012), potentially to help detect females, but this remains speculative.

### Receptor physiology differs between eye pairs

Both *O. praticola* and *X. cristatus* have the four pairs of eyes found in most spiders (Morehouse 2020). The results show that these not only increase the spiders’ visual field but also putatively serve them in different visual tasks suggested by different receptor properties. This follows the idea of special-purpose eyes (Land and Nilsson 2006), where division of labor between eyes allows for instant filtering of information, limiting the energy cost of additional processing in the brain. This is not surprising, as this has been seen in virtually all examined spiders coming from many different families with different lifestyles (Eakin and Brandenburger 1971; Blest 1977; Land and Barth 1992; Barth et al. 1993; Dacke et al. 1999; Ortega-Escobar and Munoz-Cuevas 1999; Insausti et al. 2012; Winsor et al. 2023). *X. cristatus* has its eyes divided into two distinct categories. The three secondary eyes (AL, PM, and PL) display a similar high temporal resolution with a FFF of 20–30 Hz, similar sensitivity threshold between 0.9 and 9 W/(sr ·m^2^) and a dynamic range showing no saturation at maximum intensity. The placement of these three “fast”, dynamic, and relatively sensitive pairs of eyes on the prosoma likely gives *X. cristatus* an almost 360° visual field of high temporal resolution. We speculate that this is mainly to allow detection of fast-moving predators, but possibly also to detect prey. This similarity in receptor physiology occurs despite differences in eye diameter. As discussed for the males and females of *O. praticola* we suggest that this is because the smaller eyes (PL and PM) have larger receptors and lower spatial resolution to compensate for their smaller light-capturing area.

The AM eyes of *X. cristatus* differs from the remaining eyes by having a moveable retina, but also by a low FFF of around 10 Hz, and a 2–3 log units lower absolute sensitivity. Based on what is known from jumping spiders, which are also visually guided hunters (Land 1969a; Eakin and Brandenburger 1971; Forster 1977; Lazareva et al. 2012; Winsor et al. 2023), we believe that the AM eyes have the highest spatial resolution and perhaps also color vision. High acuity color vision requires many photons, which can be obtained by lowering the temporal resolution and sensitivity. This interpretation is supported by the AM eyes of another crab spider, *Misumena vatia*, where the evidence points to these exact visual properties of the AM eyes (Insausti et al. 2012). However, we found no evidence of color vision in any of the examined eyes, which showed spectral sensitivity with a single peak at 504 nm for *X. cristatus* and 511 nm for *O. praticola*. The expression of a blue-green opsin fits well with what has been found in other spiders, including the crab spider *M. vatia* (Blest et al. 1981; Barth et al. 1993; Defrize et al. 2011). Still, it is noteworthy that we were not able to test their sensitivity in the near UV spectrum below 420 nm since there is evidence of short wavelength color vision based on a combination of a blue-green and a UV-opsin in multiple spider families, including the crab spider *M. vatia* (Blest et al. 1981; Barth et al. 1993; Lim and Li 2006; Defrize et al. 2011; DeVoe 1972, 1975).

The eyes of *O. praticola* show a high degree of specialization. The PM and AM eyes are very similar in both speed and sensitivity, with a response threshold of about 90 W/(sr ·m^2^) and a FFF around 10 Hz. As with *X. cristatus*, this could indicate that the AM eyes have high spatial resolution and possibly color vision (Foelix 2011; Insausti et al. 2012). The PM eyes are generally smaller than the AM eyes but being secondary eyes, they might have a tapetum lucidum as seen in most spiders including the PM eyes of *M. vatia* (Insausti et al. 2012; Land 1985). The increased photon capture caused by the tapetum lucidum could help compensate for the small eye diameter of the PM eyes. Little is known about the visual role of the PM eyes, but our results here show that they are probably used to detect rather large slow slow-moving objects above the spider.

The AL eyes in *O. praticola* are the largest and have their own category with a high FFF of around 20 Hz combined with a low sensitivity threshold of around 0.9 W/(sr·m^2^) and a broad dynamic range unsaturated at maximum intensities. The high FFF in these eyes could indicate two things: (1) a priority in getting visual input from fast-moving objects coming from the front of the animal with minimal motion blur, or (2) that *O. praticola* needs to avoid motion blur caused by fast self-motion. The latter is probably not the case for females, which move slowly (pers. observations), and detection of fast-moving prey and/or predators is thus a more likely function. The adult males, however, move much faster when wandering for females (pers. observations), so the high temporal resolution might also help them in mate detection. The backward-facing PL eyes are as sensitive as the AL eyes, but with a lower temporal resolution, of only about 10 Hz (similar to that seen in the two insensitive AM and PM eyes). This means that the PL eyes generate bright images of what is behind the spider but will suffer from motion blur.

### Differences between species

There are also differences in photoreceptor physiology between the two examined species, indicating different visual ecologies.

*X. cristatu*s in general has higher temporal resolution with the AL, PL, and PM eyes being faster than in *O. praticola* (FFF of 20–30 Hz vs. 10–20 Hz). The high temporal resolution fits well with *X. cristatus* being an active hunter in open habitats under high-intensity conditions. The high temporal resolution in 3 out of 4 eye pairs putatively makes the spider able to detect sudden movements of prey, movement of vegetation (because of the habitat’s general exposure to wind), and movement of predators in practically all directions, minimizing motion blur. The dynamic range of these three pairs of eyes also extends well into low intensities, suggesting that *X. cristatu*s can perform visual hunting and navigation even at dusk and dawn. However, the lower light intensity at that time of day will probably have a significant effect on the eye’s temporal resolution and thereby affect the “effectiveness” of visually guided behavior (Land and Nilsson 2012).

The eye physiology of *O. praticola* is quite different, with low temporal resolution in 3 out of 4 eyes (AM, PM, and PL) and high sensitivity in two sets (AL and PL). The forward-directed AL eyes are more or less similar in sensitivity to *X. cristatus*, while the PL eyes seem to be more sensitive in *O. praticola* compared to *X. cristatus*, with the *V*-Log *I* curve extending far into low intensities with a sensitivity threshold of about 0.2 W/(sr ·m^2^). The data for this set of eyes are noisy and only based on males, though, adding some uncertainty to this result. The AM eyes of *O. praticola* also appear slightly more sensitive than in *X. cristatus* with a *V*-Log *I* curve shifted about 0.5–1.5 log unit towards lower intensities in *O. praticola*. These similar or slightly higher sensitivities in the AL, AM, and PL eyes of *O. praticola* compared to *X. cristatus*, are interesting since the eyes of *X. cristatus* are larger in general with a larger area for photon capture. One explanation for this could be that *O. praticola* has similar sensitivity adaptations as those seen in nocturnal spiders like *Cupiennius salei* (Trechaleidae). Such adaptations could be longer integration times (which would explain the lower temporal resolution in the PL eyes), but also structural elements like longer photoreceptors and a lens with a smaller f-number, at the cost of spatial resolution (Land and Barth 1992; Barth et al. 1993; Grusch et al. 1997).

The tendency to high sensitivity of the eyes in *O. praticola* (despite smaller apertures) is a good match with the dim leaf litter and crevices that *O. praticola* inhabits. In such habitats with low light intensities, photoreceptors with high sensitivity should be favored. That *O. praticola*, despite its dim habitat, is still a visually guided animal is seen by the high temporal resolution of the forward-directed AL eyes, which are probably used for prey capture as in many other visually guided spiders (Land 1969a; Blest 1977).

Considering its rather dark habitat, it is surprising that the eyes of *O. praticola* did not saturate at full light intensity. This contradicts our hypothesis that this species would have a limited dynamic range shifted to lower light intensities. The authors have observational evidence that subadult males of *O. praticola* can be active at nighttime. They were observed wandering at night three times, still one adult male was seen wandering in bright daylight, and another adult male wandered in daylight but in the shade. It is difficult to assess the activity patterns of females, as they mostly sit still and are harder to spot. However, if timestamps are correct, three images captured in the afternoon of females (Fig. S2) show feeding activity (captured: 13:31, 15:38 & 16:01 respectively). This and the adult male’s daylight wandering indicate that the species can be active at times with greater light intensities, which may explain its broader dynamic range extending well into high light intensities in all their eyes.

Assuming *O. praticola* is a visually guided predator, the low temporal resolution in the AM, PM, and PL eyes (FFF 10–11 Hz), may be linked to prey preference. Observational evidence (Fig S2) indicates slow-moving springtails being part of their diet – very different from the large, fast, and flying insects that *X. cristatus* feeds on (Fig. S1). The leaf litter habitat of *O. praticola*, putatively minimizes the predation risk from predators like birds, and this could also be part of the explanation. It is often seen that *X. cristatus* drops to the ground when you approach them (pers. observations), a means of escape that requires a fast detection of sudden incoming movement.

The PM eyes show the largest difference between the two species. In *O. praticola* they have a low temporal resolution of 11 Hz and low sensitivity (response threshold of 90 W/(sr ·m^2^), which is very different from *X. cristatus* where the PM eyes have a high temporal resolution of 20 Hz and a 2 log units higher sensitivity. A hypothesis is that since they look upwards, the PM eyes in *O. praticola* might simply be light sensors giving information on light intensities and general habitat structures, signaling whether the spider is in its preferred niche. The PM eyes in *X. cristatus*, on the contrary, might, as previously mentioned, also have an important role in evading predation from flying predators like birds, which requires the spider’s eyes to be fast and sensitive.

## Supplementary Information

Below is the link to the electronic supplementary material.

Supplementary Material 1

## Data Availability

Data can be supplied on request.
