# Supplementary material for: Photoreceptor physiology of two species of crab spiders (Araneae: Thomisidae)
Source: J Comp Physiol A Neuroethol Sens Neural Behav Physiol. 2026 Mar 24;212(4):579–91. doi: 10.1007/s00359-026-01802-8 (PMC13395977; doi:10.1007/s00359-026-01802-8)
Supplement: Supplementary file 1 — Supplementary Material 1 [file 359_2026_1802_MOESM1_ESM.docx]

**Supplementary material**


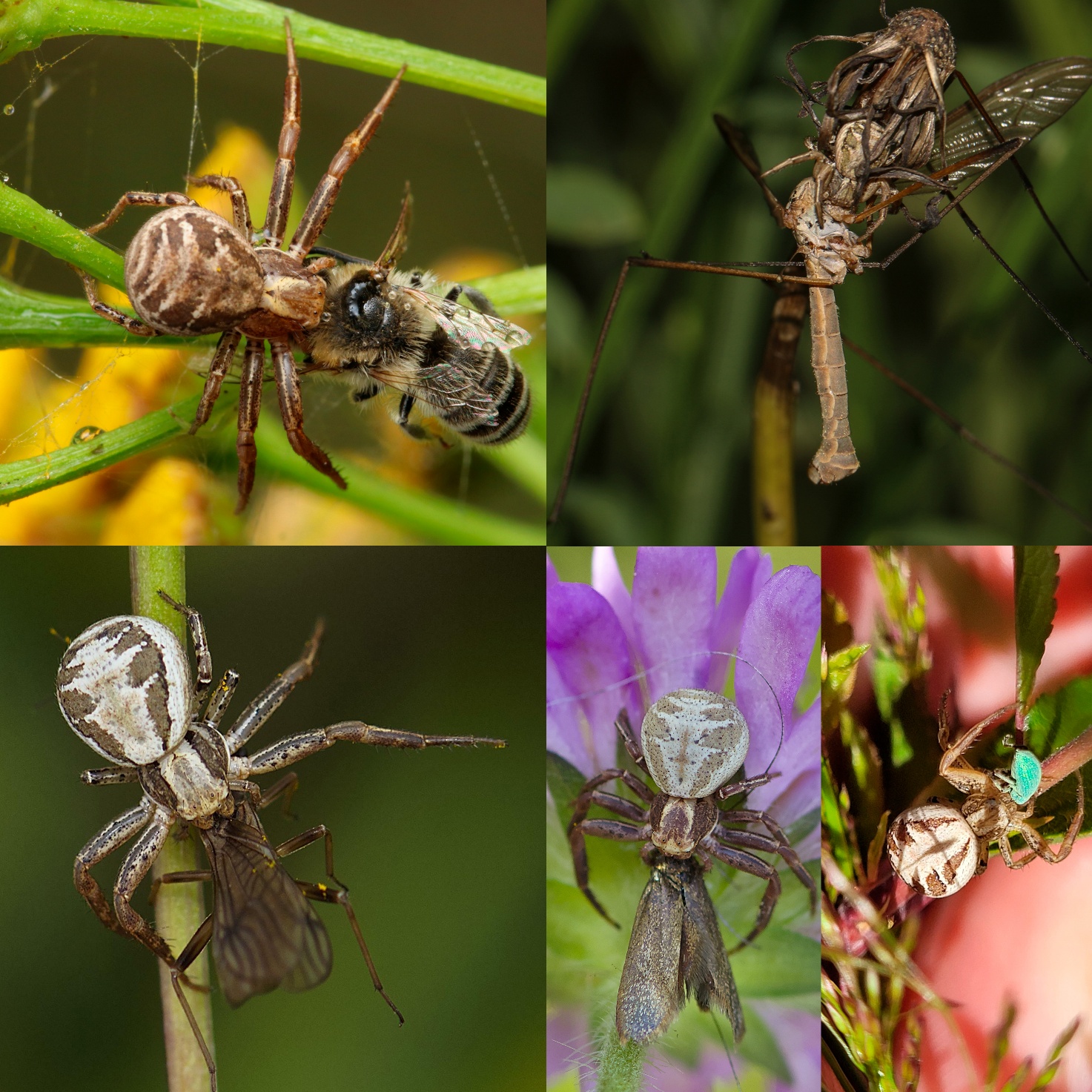


**Figure S1** – Examples of the diet of female *Xysticus cristatus*, top left: A bee in genus *Colletes*, top right: A cranefly (Tipulidae), Bottom row from left to right: unknown dipteran, unknown moth, weevil likely in the genus *Polydrusus*. Image credits: Fabian A. Boetzl <https://www.inaturalist.org/observations/175342272#activity_comment_199f23bb-8b99-4bc7-bfae-c51af08817ea>, Frederik Leck Fischer, Bøje Rauff <https://www.inaturalist.org/observations/229911434#activity_comment_3f278d80-2108-44d9-a72a-77078a2bd1da> & <https://www.inaturalist.org/observations/188664989#activity_comment_06381fb1-477b-4799-abb4-82ba7d777595>, Linda Larsen <https://www.inaturalist.org/observations/223784201>


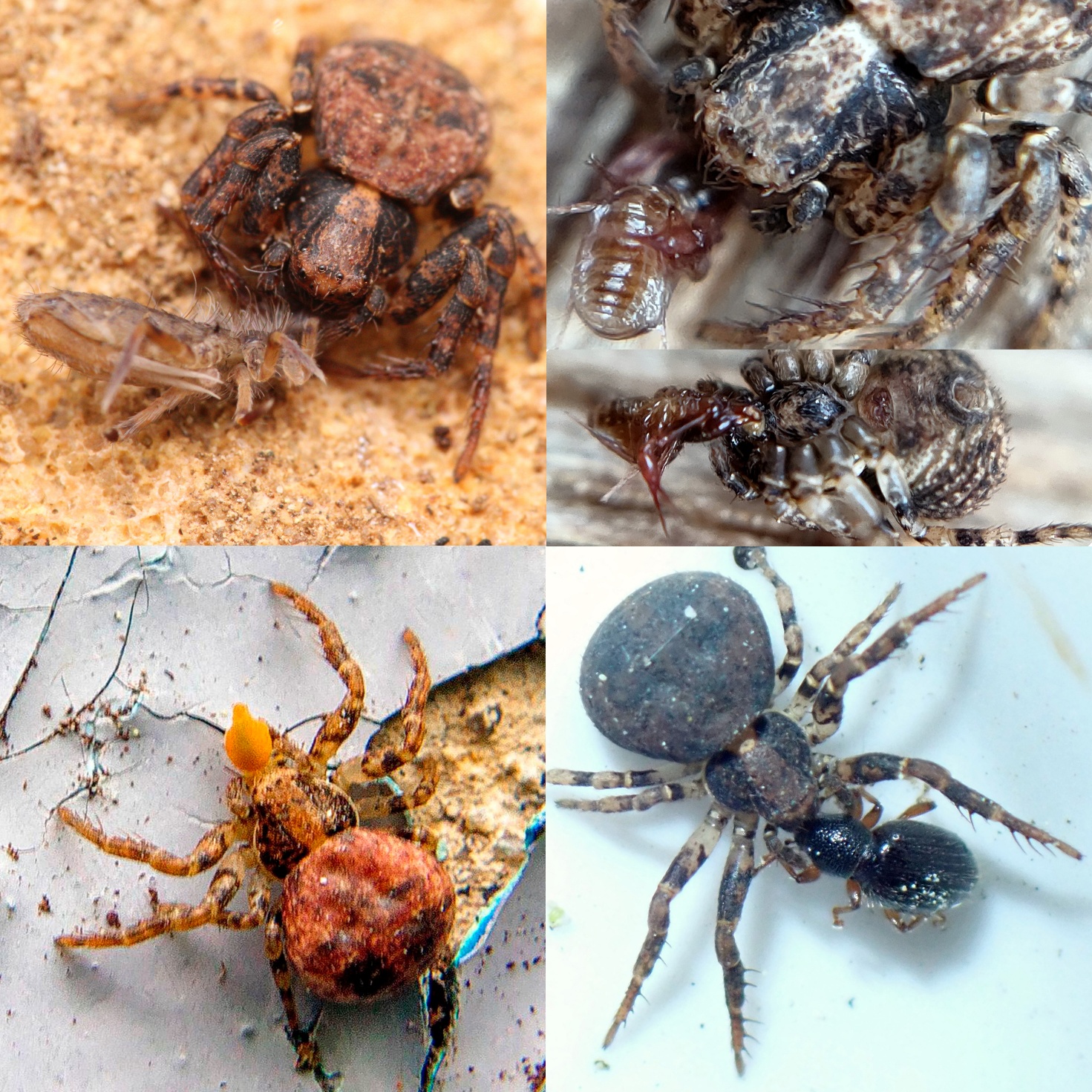


**Figure S2** – Images showing the diet of female *Ozyptila praticola*, top left: Springtail in family Tomoceridae, top right: Pseudoscorpion in genus *Neobisium*, Bottom left: Springtail, possibly in genus *Sminthurus*, Bottom right: Weevil in the genus *Exomias* . Image credits: Shreyas Kuchibhotla <https://www.inaturalist.org/observations/198229725>, iNaturalist user Jfb22: <https://www.inaturalist.org/observations/239974037>, René Faucher <https://www.inaturalist.org/observations/28208745> , Linda Kjær-Thomsen <https://arter.dk/observation/record-details/5edd3204-18c0-4ff5-be97-b1af0142a2d7>


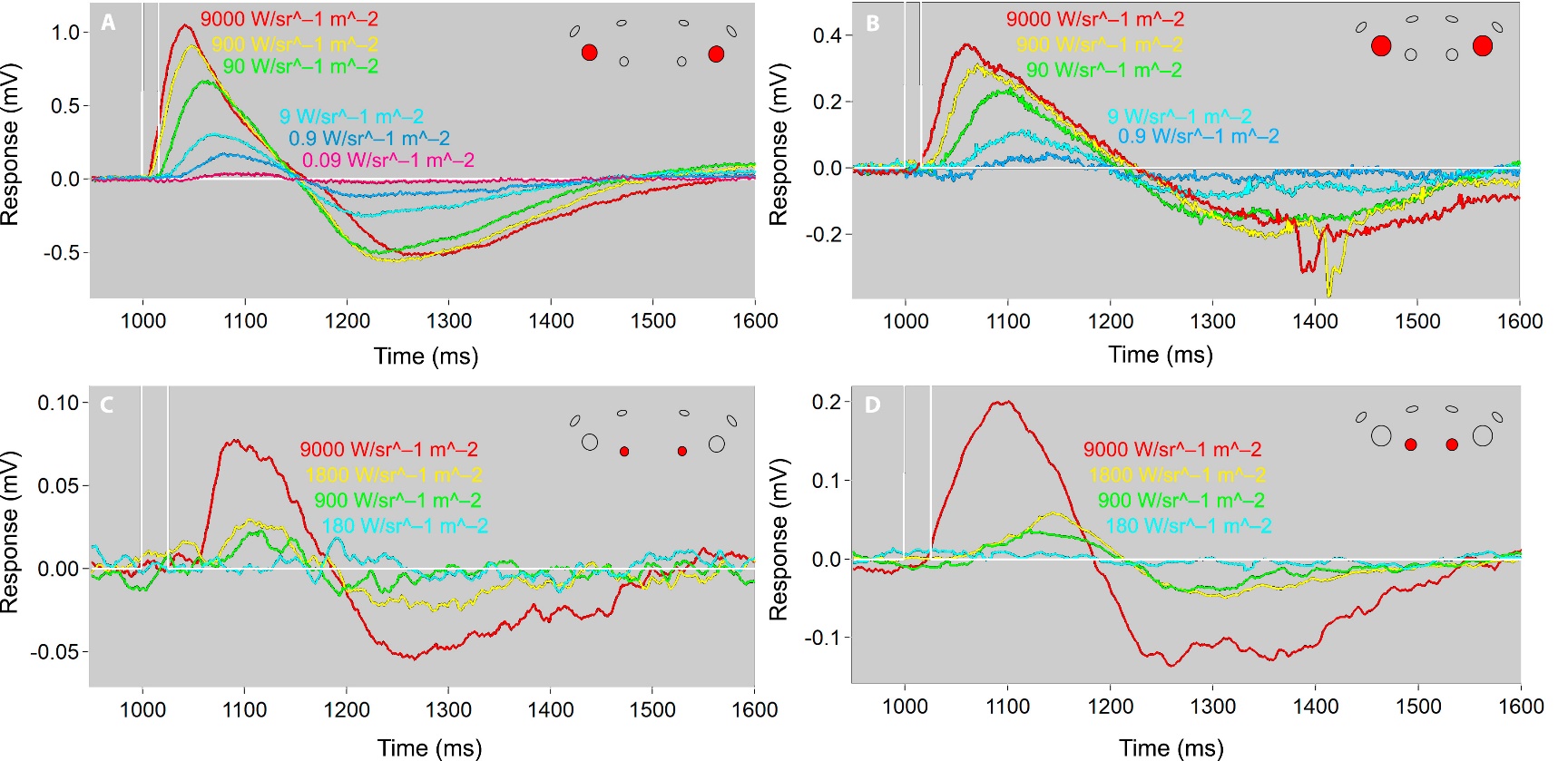


**Figure S3** – Examples of V-log I series from 2 eye types. A) *Xysticus cristatus* anterior lateral eyes, B) *Ozyptila praticola* anterior lateral eyes C) *X. cristatus* anterior median eyes, D) *O. praticola* Anterior median eyes. Note the weaker and more noisy response from the smaller AM eyes in both species despite them being filtered with a running average of 9 data points.


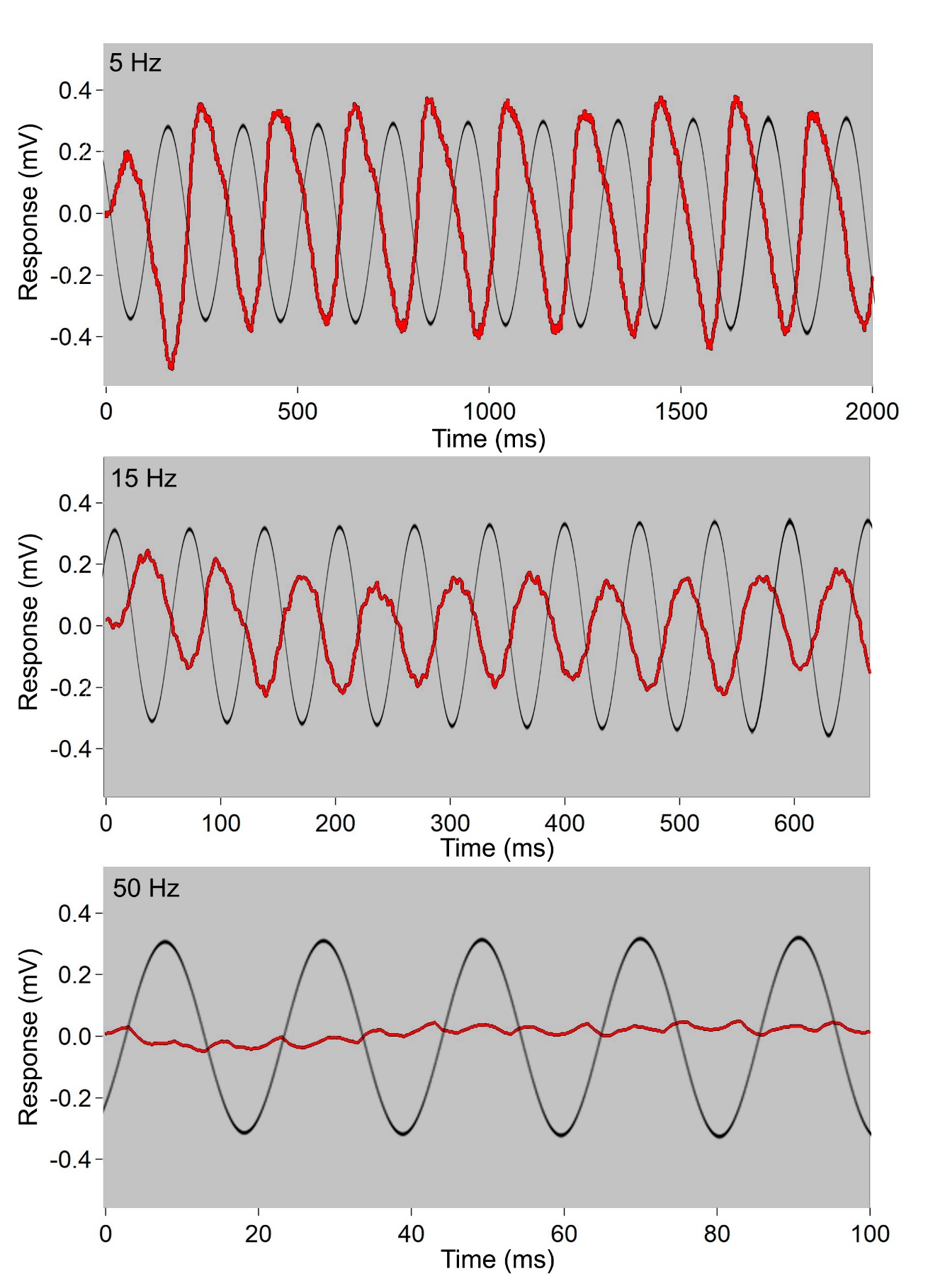


**Figure S4** – Examples of FFF measurements (red line) at different Hertz (5, 15, and 50 hz) of the anterior lateral eye of an *X. cristatus* female.


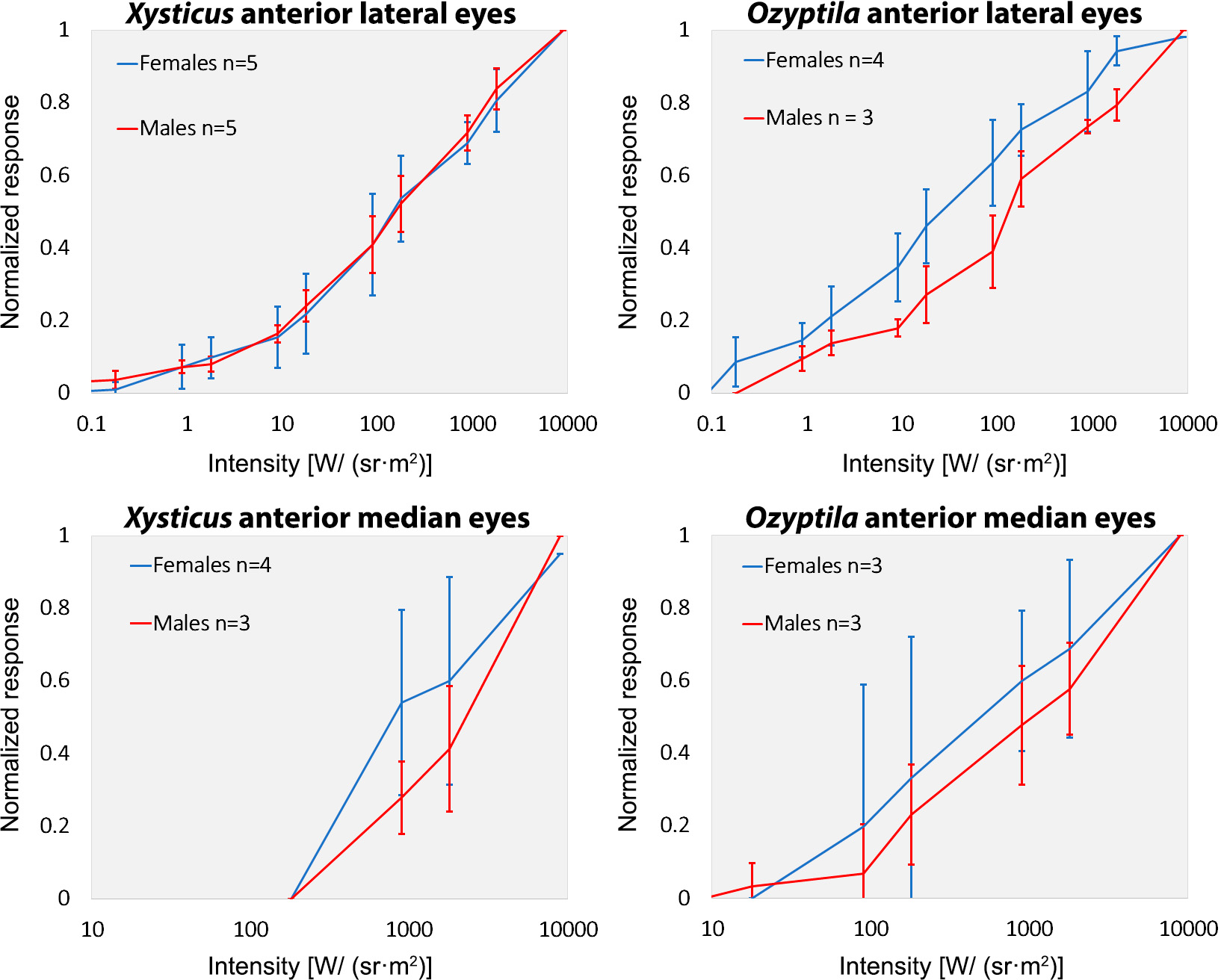


**Figure S5 –** Examples of absolute sensitivity (V-log I curves) of AM and AL eyes of males and females of both species. In *Xysticus* the AL and AM are very similar for the sexes where as in *Ozyptila* there is a weak tendency for the male to be less sensitive. Note that the x-axis is logarithmic. Error bars indicate 95% confidence intervals.


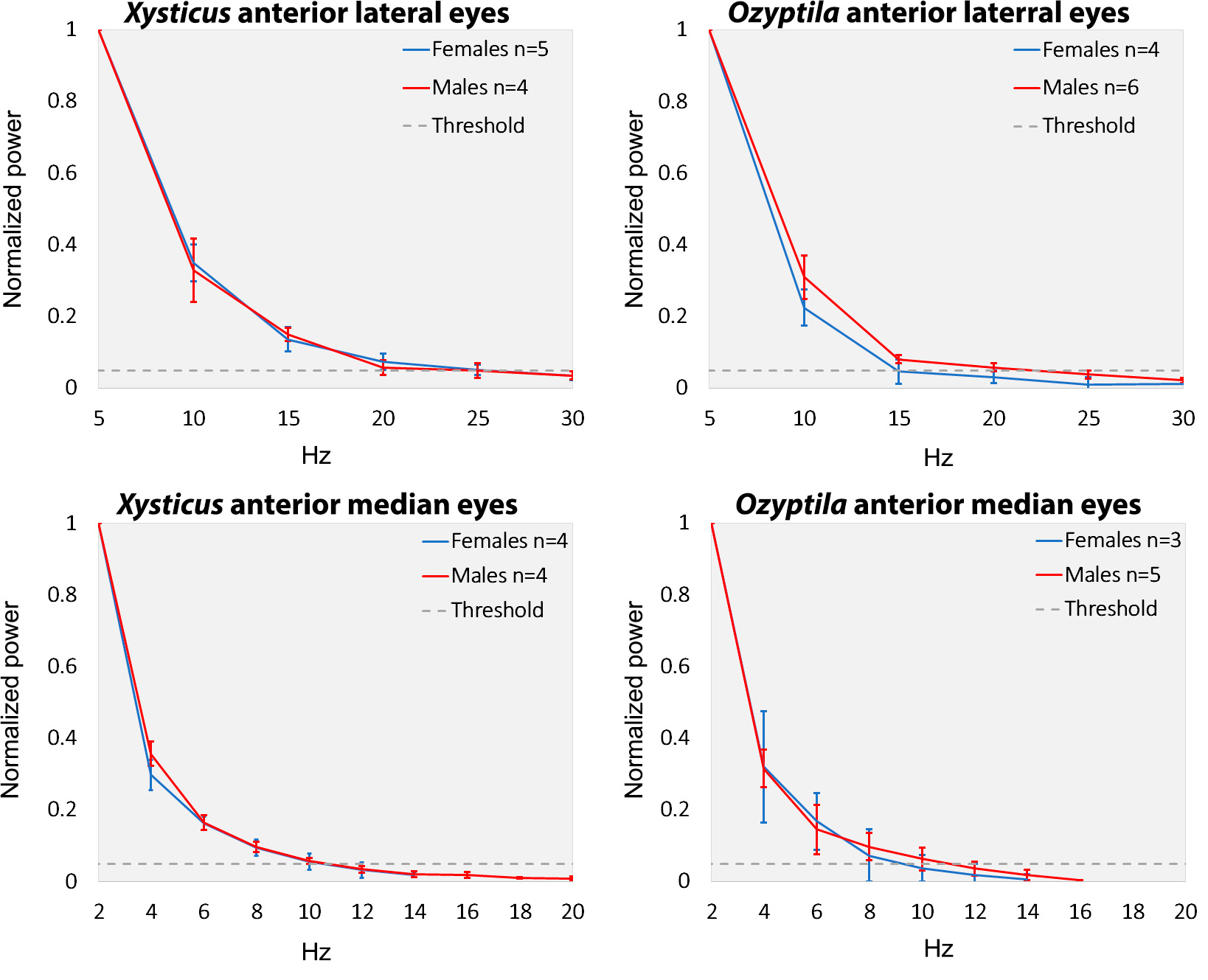


**Figure S6 -** Examples of temporal resolution (FFF curves) of AL and AM eyes of males and females of both species. Threshold/FFF is defined as 5% of the maximum power. Note that both eyes have very similar temporal resolution for the 2 sexes in both species. Error bars indicate 95% confidence intervals.


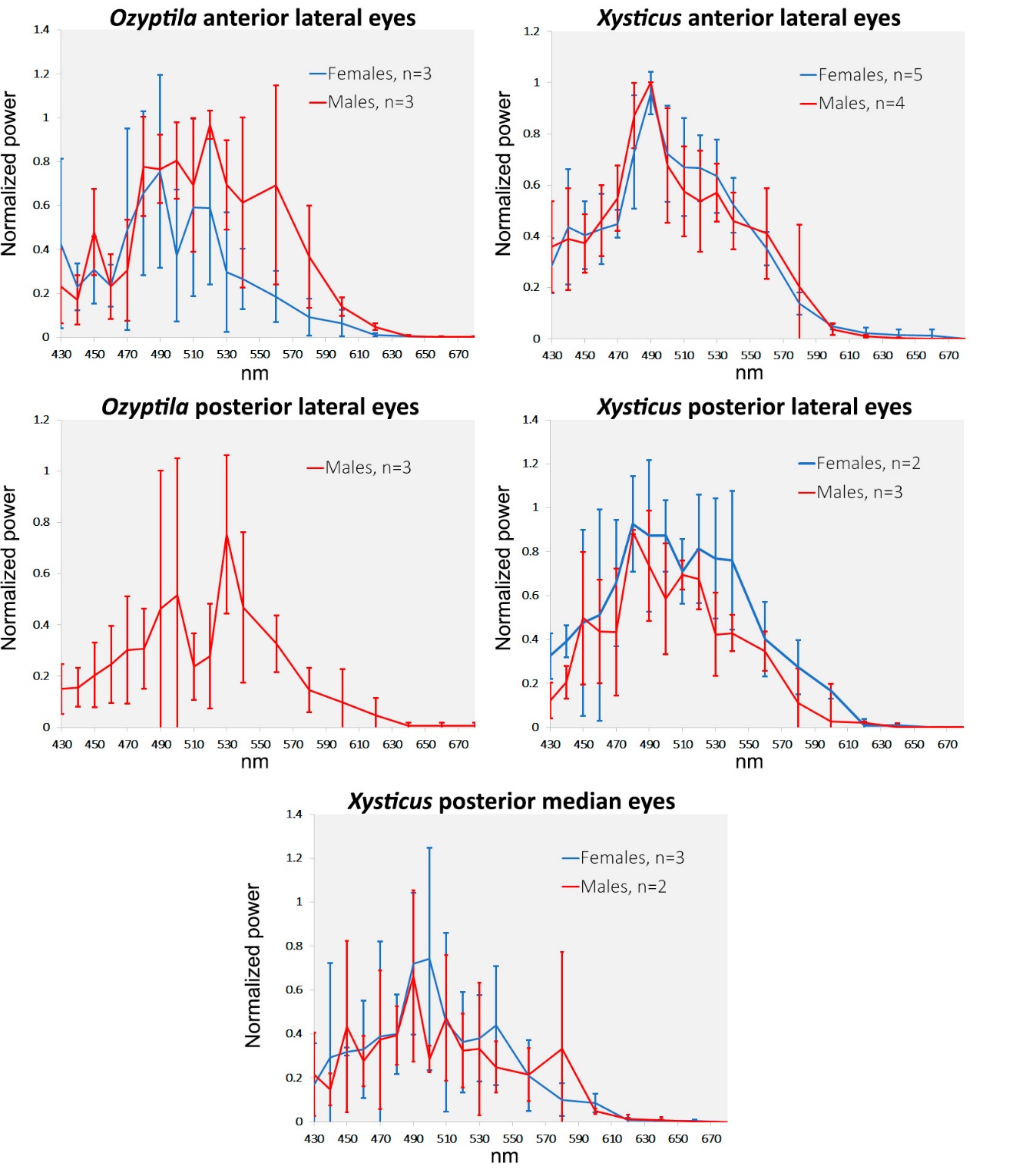


**Figure S7** – Examples of raw spectral sensitivity data of 3 eyes, with comparison of male and female. Error bars indicate 95% confidence intervals.
